# Found in transition: applying milestones to three unique discharge curricula

**DOI:** 10.7717/peerj.819

**Published:** 2015-03-05

**Authors:** Lauren B. Meade, Christine Y. Todd, Meghan M. Walsh

**Affiliations:** 1 Macy Faculty Scholar, Baystate Medical Center, Tufts University Medical School, Springfield, MA, USA; 2 Department of Medical Humanities, Southern Illinois University, Springfield, IL, USA; 3 Transitional Residency Program, Hennepin County Medical Center, Minneapolis, MN, USA

**Keywords:** Transition of care, Milestones, Entrustable professional activities, Medical education, Discharge, Core competence

## Abstract

**Introduction.** A safe and effective transition from hospital to post-acute care is a complex and important physician competency. Milestones and Entrustable Professional Activities (EPA) form the new educational rubric in Graduate Medical Education Training. “A safe and effective discharge from the hospital” is an EPA ripe for educational innovation.

**Methods.** The authors collaborated in a qualitative process called *mapping* to define 22 of 142 Internal Medicine (IM) curricular milestones related to the transition of care. Fifty-five participant units at an Association for Program Directors in Internal Medicine (APDIM) workshop prioritized the milestones, using a validated ranking process called Q-sort. We analyzed the Q-sort results, which rank the milestones in order of priority. We then applied this ranking to three innovative models of training IM residents in the transitions of care: Simulation (S), Discharge Clinic Feedback (DCF) and TRACER (T).

**Results.** We collected 55 Q-sort rankings from particpants at the APDIM workshop. We then identified which milestones are a focus of the three innovative models of training in the transition of care: Simulation = 5 of 22 milestones, Discharge Clinic Feedback = 9 of 22 milestones, and TRACER = 7 of 22 milestones. Milestones identified in each innovation related to one of the top 8 prioritized milestones 75% of the time; thus, more frequently than the milestones with lower priority. Two milestones are shared by all three curricula: Utilize patient-centered education and Ensure succinct written communication. Two other milestones are shared by two curricula: Manage and coordinate care transitions across multiple delivery systems and Customize care in the context of the patient’s preferences. If you combine the three innovations, all of the top 8 milestones are included.

**Discussion.** The milestones give us a context to share individual innovations and to compare and contrast using a standardized frame. We demonstrate that the three unique discharge curricula in aggregate capture all of the highest prioritized milestones for this discharge EPA.

## Introduction

The ability to safely and effectively manage transitions of care represents a critical new competency for today’s internist. Brought to light by the increasingly prevalent separation between inpatient and outpatient services, the medical literature continues to demonstrate that poorly managed transitions of care result in significant medical errors, decreased patient satisfaction, and poor patient outcomes (Jencks, Williams & Coleman, 2009; Van Walraven et al., 2002; Kripalani et al., 2007; Forster et al., 2003). Coleman & Berenson (2004) describe the fragmentation of care in a landmark paper with the ominous title, “Lost in Transition.”

While practicing internists attempt to master their own transfer of care skills, the academic medical community is challenged by the need to identify and incorporate these skills into an effective transition of care curriculum for trainees. Discharging patients from the hospital, a major transition of care event, is a daily occurrence in the lives of Internal Medicine house-staff, yet medical literature guiding the training, supervision, and evaluation of this critical event is limited. Several studies have highlighted innovative curriculum surrounding transitions of care. Self-audits of resident discharge summaries (Dinescu et al., 2011; Talwalkar et al., 2012) and feedback about the discharge summary (Myers et al., 2006; Legault et al., 2012) improved discharge summary targets. A discharge “time out” (Coit, Katz & McMahon, 2011) and reduced house-staff workload also improved discharge summaries (Mohta et al., 2012). A web-based module utilizing a well-designed teaching case emphasized the importance of communication in the transition (Eskildsen, 2010). A qualitative study of the resident perspective on the interdisciplinary nature of teamwork revealed “learning by doing” as the foremost theme in two residency programs (Greysen et al., 2012). A medical student curriculum using experiential learning that follows a patient through the transition improved confidence (Lai et al., 2008) and was found to be both feasible and effective (Bray-Hall, Schmidt & Aagaard, 2010).

Beyond the challenge of developing a program for training physicians in a new patient care venue, academic internists are facing increasingly rigorous standards for measureable training outcomes. Milestones and Entrustable Professional Activities (EPAs) are an evolving framework for defining the competence of medical trainees. Milestones are discrete observable behaviors that demonstrate competence (Green et al., 2009). An EPA is an activity that constitutes the mass of critical elements or knowledge, skills, and attitudes (KSAs) that operationally define a profession (Ten Cate & Scheele, 2007; Hauer et al., 2013). The theory of milestones and EPAs have been well described and are beginning to be adopted as best practice goals for Internal Medicine (IM) residency programs, but there are few examples in the medical literature illustrating how to effectively turn this educational theory into practice (Nasca et al., 2012; Schumacher, Englander & Carraccio, 2013).

The KSAs needed to accomplish a high quality discharge are both complex and challenging, thus ripe for the application of this new paradigm of milestones and EPAs. The authors of this report created three unique curricula in transitions of care training of medical resident in their separate institutions prior to 2009, in other words, prior to the publication of the curricular milestones (Green et al., 2009). With a call for using milestones for standard setting, we embarked on a standard-setting process for applying milestones to our three transitions of care curricula across institutions.

In 2011, the authors presented an Association for Program Directors of Internal Medicine (APDIM) workshop on the transition of care (Meade, Walsh & Todd, 2011) both to describe the individual transitions of care at our institutions and to discuss and collect data on workshop participants’ prioritization of milestones for transitions of care and specifically the EPA, “A safe and effective discharge from the hospital.” After analyzing the Q-sort data, we reflected on our original curricula, using the prioritized milestones as a gold standard to compare and contrast our transition of care program curriculum. This report describes three curricula in the transition of care, the results of the group Q-sort, and the application of the prioritized milestones back to the curricula as a way to compare and contrast curriculum in for training in an EPA.

## Methods

### Mapping milestones

The authors collaborated in a qualitative process called mapping, to develop a Q-sort exercise for workshop participants related to the EPA, “A safe and effective discharge from the hospital.” First, we individually choose 22 milestones of the 142 curricular milestones that applied to the EPA. Then, over three months, we discussed and deliberated our choices in the group process, in three rounds, until there was agreement on the 22 curricular milestones most related to the EPA. Table 1 lists the 22 milestones using an abbreviated nomenclature based on core competence and domain, for example, “effectively communicates with other caregivers during transitions of care” is an Interprofessional Communication (IPC) core competence of domain C and item 1 (C1) (Green et al., 2009). Using these 22 milestones, we created a Q-sort exercise to distribute and collect at an APDIM workshop (Meade et al., 2013). For the exercise we created cards with a curricular milestone on the front and an example of the milestones as it applies to the EPA on the back. In a similar iterative process, we also mapped curricular milestones to our individual curricula.

**Table 1:** Milestones for the EPA “A safe and effective discharge.” Lists the 22 Internal Medicine Curricular milestones by category competency/domain (Column A); the Q-sort priority for the milestones (Column B) from highest to lowest with 7, the highest priority and 1, the lowest priority; the standard deviation of prioritization (Column C); the milestones descriptor (Column D), the transition of care example for that milestone (Column E) and an X to denote if this milestones was present in the four categories (Column F–I) of top 8 in Q-sort (top 8), Simulation (S), Discharge clinic feedback (DCF), Tracer (T).

	Priority	SD	Milestones abbreviated	Example	Top 8 Q-sort	S	DCF	T
IPC C1*	5.78	0.97	Effectively communicate with other caregivers during transitions of care	Communicates with the PCP or nursing home at discharge.	**X**			**X**
SBP A2	5.52	1.16	Manage and coordinate care and care transitions across multiple delivery systems.	Works with the case manager to make appropriate decisions about where a patient should go after discharge ie home with nursing services or to a nursing home.	**X**		**X**	**X**
PC C1	4.89	1.25	Synthesize all available data	Able to synthesize a complicated hospital course.	**X**	**X**		
IPC D3	4.74	1.35	Engage in collaborative communication with all members of the health care team	Seeks out the nurse and case manager about the plan for discharge.	**X**			**X**
IPC A5	4.56	1.63	Utilize patient-centered education strategies	Explains the primary diagnosis to the patient at discharge and uses teach back to check for understanding.	**X**	**X**	**X**	**X**
IPC F2	4.48	1.31	Ensure succinct, relevant, and patient-specific written communication	A succinct discharge summary with key components.	**X**	**X**	**X**	**X**
P A1	4.48	1.85	Document and report clinical information truthfully	Really did call the pharmacy to confirm the medication list if they say they have.	**X**	**X**		
PC F10	4.26	1.20	Customize care in the context of the patient’s preferences and overall health	Offers home care or nursing home care depending on patient preferences.	**X**		**X**	**X**
PC C3	4.15	1.17	Modify differential diagnosis and care plan based upon clinical course and data as appropriate	If the patient is admitted with presumed pnemonia but the clinical course is consistent with CHF then this resident identifies CHF as the final diagnosis and is able to explain why it is not pneumonia.			**X**	
P i1	4.15	1.29	Treat patients with dignity, civility and respect, regardless of race, culture, gender, ethnicity, age or socioeconomic status	Makes special accommodations for a homeless patient such as having social work assist with clothing, food and/or shelter.				
PC B2	3.96	1.22	Accurately track important changes in the physical examination	Documents the mental status physical exam upon discharge for a patient admitted with altered mental status.			**X**	
PC A2	3.93	1.17	Seek and obtain appropriate, verified, and prioritized data from secondary sources	Verifies the medication list with the pharmacy or PCP.				
P D2	3.74	1.16	Carry out timely interactions with colleagues, patients and their designated caregivers	Completes the discharge summary within 24 h of discharge.			**X**	
MK A9	3.70	1.14	Demonstrate sufficient knowledge of socio-behavioral sciences	Has the knowledge that a patient without health insurance may have many barriers to transition of care such as cost of medications, access to PCP, and poor health literacy.				
IPC A4	3.70	1.27	Engage patients/advocates in shared decision-making for uncomplicated diagnostic and therapeutic scenarios	Checks with the patient about the convenience of the follow up appointment.				
SBP B1	3.58	1.30	Appreciate roles of a variety of health care providers	Uses the home nurse for to assist with education of the primary diagnosis and medication reconciliation after discharge.		**X**		**X**
PBLI F1	3.50	1.17	Respond welcomingly and productively to feedback from all members of the health care team	Responds to nursing concerns about readiness for discharge.			**X**	
IPC E3	3.50	1.21	Communicate consultative recommendations to the referring team in an effective manner	Includes the name and recommendations of a consultant in the discharge summary.				
SBP E3	3.41	1.47	Demonstrate the incorporation of cost-awareness principles	Uses the antibiotic that is most appropriate but also affordable to the outpatient at discharge.				
P J1	3.11	1.45	Maintain patient confidentiality	Knows to get permission from the patient or their health care proxy to disclose any medical information.				
P F7	2.54	1.27	Recognize the need to assist colleagues in the provision of duties	A supervising resident who does a discharge for an intern because it is too complex for that intern.				
PBLI A3	2.41	1.12	Reflect on audit compared with local or national benchmarks	Is aware of the high risk concerns for re-admission.			**X**	

**Notes.**

IPC Interpersonal communication SBP Systems based practice PC Patient care P Professionalism MK Medical knowledge PBLI Problem-based learning and improvement

### Prioritizing milestones

We distributed the Q-sort exercise, a validated ranking process, for this EPA to participants at the APDIM workshop. Participants were IM program faculty who worked in groups of two to three, called participant units, to prioritize the milestones for the EPA. Participants were given instructions on how to rank the milestones by Q-sort methodology. Q-sort methodology enables researchers with some limitation to study subjectivity using a combination of qualitative and quantitative methods (Brown, Danielson & Van Exel, 2014). It prioritizes the opinions of an observer (Brown, 1996) and provides an organized means of identifying priorities and areas of divergent opinions within a group (Valenta & Wigger, 1997). In a Q-sort, the observer rank-orders a set of statements from most important to least important, using an inverted quasi-normal distribution (Brown, 1996; Van Exel & deGraaf, 2005). The sample of statements (the Q sample) may represent an existing framework, in this case, the 22 milestones related to the EPA (Brown, 1996; Van Exel & deGraaf, 2005). Thus, the statements are the unit of analysis; the number of observers is less important than is their theoretical relevance to the topic (Valenta & Wigger, 1997). Once sorted, statements are analyzed by rank category with a standard deviation. The Q-sort method uses a mathematical substructure to reveal priorities of subjective viewpoints of the observers. The results of the Q-sort can be used to describe the sample of viewpoints, in this case, in prioritization of milestones, rather than the sample of observers (Van Exel & deGraaf, 2005).

We analyzed the Q-sort results by calculating the mean rank order of milestones by faculty units. With this mean ranking, we listed the milestones in order of priority. We identified the top 8 milestones because, by Q-sort methodology, the top 8 milestones confer those milestones more than neutral status in the normal distribution. We calculated the proportion of milestones used of the total 22 milestones in the curriculum for each program. With each innovation, we calculated the proportion of milestones that were in the top 8 compared to the total. We then considered the milestones in common between curricula both in the total 22 milestones and in the top 8 milestones to look for trends.

## Results

First, we will describe the milestones as they were mapped to each transitions of care program. Then, we will describe the Q-sort exercise results. Finally, we will reflect on our individual curricula using the prioritized milestones as a standard for curriculum.

### Mapping milestones

In this section we will briefly describe the individual transitions of care curricula and the results of mapping the milestones to each program.

### Simulation

SIU is a small university program in Springfield, Illinois with one academic ambulatory site and two affiliate hospitals. During an Objective Structured Systems-Interaction Exam or simulation, residents are observed discharging a patient from the hospital and evaluated on how well they demonstrated the following observable behaviors: Use of the Situation, Background, Assessment, Recommendation format to notify the Anti-Coagulation clinic of the patient’s Warfarin levels and dose, Use of Electronic Health Record to document medication changes, Use of the Hospital’s medication reconciliation form, and Legibility and Accuracy of written prescriptions. Five of the 22 curricular milestones are identified for the simulation curriculum: Patient Care (PC) C1, Synthesize all available data; IPC A5, Utilize patient-centered education strategies; IPC F2, Ensure succinct, relevant, and patient-specific written communication; Professionalism (P) A1, Document and report clinical information truthfully; System-Based Practice (SBP) B1, Appreciate roles of a variety of health care providers.

### Discharge clinic feedback

Hennepin County Medical Center is a moderate-sized academic training program in Minneapolis, Minnesota with one ambulatory clinic and one hospital affiliate. This innovation targets the nine primary care track residents in the program. A discharge clinic feedback provides patients with pharmacist-assisted medication reconciliation, lab and radiology testing follow-up, and appointment confirmation. The resident evaluates the quality of the discharge and gives feedback to the ward team resident who discharged the patient. This evaluation includes direct feedback from the patient regarding the discharge processes as well as structured feedback on the written discharge summary. Nine of the 22 curricular milestones are identified for the discharge clinic feedback curriculum: SBP A2, Manage and coordinate care and care transitions across multiple delivery systems; IPC A5, Utilize patient-centered education strategies; IPC F2, Ensure succinct, relevant, and patient-specific written communication; PC F10, Customize care in the context of the patient’s preferences and overall health; PC C3, Modify differential diagnosis and care plan based upon clinical course and data as appropriate; PC B2, Accurately track important changes in the physical examination; P D2, Carry out timely interactions with colleagues, patients and their designated caregivers; Problem-Based Learning and Improvement (PBLI) F1, Respond welcomingly and productively to feedback from all members of the health care team; PBLI A3, Reflect on audit compared with local or national benchmarks.

### Tracer

Baystate Medical Center, Springfield, Massachusetts, is a moderate-sized, academic training program with one academic ambulatory site and one large hospital affiliate. In a two-week experiential block rotation, the TRAnsitions of Care Rotation (TRACER) resident follows the ward team patient into the Post-Acute Care (PAC) settings, including home, rehabilitation, and long-term care. Follow-up includes a visit to the patient in PAC and a formal assessment of the transition, using tools modified from the transition of care literature. These data are communicated in aggregate to the hospital PAC Performance Improvement team, to the ward team, at inter-professional attending rounds, and at morning report. Seven curricular milestones are identified for the Tracer curriculum: IPC C1, Effectively communicate with other caregivers during transitions of care; SBP A2, Manage and coordinate care and care transitions across multiple delivery systems; IPC D3, Engage in collaborative communication with all members of the health care team; IPC A5, Utilize patient-centered education strategies; IPC F2, Ensure succinct, relevant, and patient-specific written communication; PC F10, Customize care in the context of the patient’s preferences and overall health; SBP B1, Appreciate roles of a variety of health care providers.

### Prioritizing and applying milestones

We collected 55 Q-sort rankings from faculty units at the APDIM workshop who ranked the 22 IM curricular milestones related to “a safe and effective discharge form the hospital.” We report the prioritized milestones by Q-sort from the APDIM workshop (Table 1) on transitions of care. The priority range for the top 8 prioritized milestones is 4.2–5.7 (SD 0.97–1.84). From the total 22 milestones, the simulation innovation identified 5 of 22 milestones, discharge clinic 9 of 22 milestones, and tracer 7 of 22 milestones related to the EPA.

We considered two tiers of milestones. We emphasize the top 8 milestones in our analysis for reasons described in the methods. Milestones identified in each innovation related to one of the top 8 prioritized milestones 75% of the time; thus, more frequently than the milestones with lower priority. Four of the top 8 milestones were shared by curricula. Two milestones are shared by all three curricula: IPC A5, Utilize patient-centered education and IPC F2, Ensure succinct, relevant, and patient-specific written communication. Two other milestones are shared by two curricula: SBP A2, Manage and coordinate care and care transitions across multiple delivery systems and PC F10, Customize care in the context of the patient’s preferences and overall health. Alternatively, few milestones are in the lower priority milestones set, and there is only 1 milestone shared by more than a single program in the lower priority milestones, that is, SBP B1, Appreciate roles of a variety of health care providers, including, but not limited to, consultants, therapists, nurses, home care workers, pharmacists, and social workers. If you combine the three innovations, all of the top 8 milestones are included.

## Conclusion

In a learning community of innovative educators, we identified the transition of care curriculum as important, largely lacking, and challenging to develop. Three curricula mapped their individual program innovations to the new assessment framework of milestones related to the EPA, “A safe and effective discharge from the hospital.” We found that the milestones in our individual curricula had a strong correlation with higher prioritized milestones in the EPA. Given this correlation, prioritizing IM curriculum milestones in an iterative process may have application in medical education curriculum development, especially across programs.

A limitation of this collaboration is its retrospective look at innovation in a new framework. The retrospective aspect is a historical phenomenon; that is, these curricula existed prior to the milestones framework. Although non-traditional, this retrospective look allows for an inquisitive matching of milestones across program innovation. Another limit is that the authors each rated the milestones associated with their own program without a peer or participant checker or a group vetting process. Nonetheless, there is no other literature in comparing innovative curricula in a specific EPA in the context of the milestones. This work is useful for future prospective work on educational assessment of the Transition of Care.

The transition of care remains a challenge to the patient, to the health care system, and to training programs. Tools related to the assessment of “a safe and effective discharge from the hospital” help narrow the current training gap. Programs may apply one or all of the transitions of care curricula described. Alternatively, programs may consider applying the top 8 milestones defined by the prioritization method, Q-sort, to an already existing program in transition of care, thereby assessing the relevance of their curriculum to a benchmark. Establishing a list of milestones for an EPA can occur in siloes at each individual program or in collaborative groups. The Q-sort method may have a role in the process as we develop standards for EPAs in medical education. Milestones are a new training rubric for measurable outcomes and competency-based advancement. We need innovative ways to apply milestones and EPAs to curricula (Hauer et al., 2013). The milestones give us a context to share individual innovations and to compare and contrast using a standardized frame.
